# Account of Diseases in an Eastern District of London, from the 20th of July to the 20th of August, 1800

**Published:** 1800-09

**Authors:** 


*Account of Diseases in an Eastern District of London, from the 20th of July to the 20th of August,* 1800.

| | No. of Cases. | | No. of Cases. |
|---|---|---|---|
| ACUTE DISEASES. | | Hemiplegia | 2 |
| Typhus | 12 | Chorea | 1 |
| Ephemera | 2 | Lumbago | 1 |
| Dysentery | 6 | Sciatica | 1 |
| Cholera Morbus | 15 | Chronic Rheumatism | 6 |
| CHRONIC DISEASES. | | Psora | 2 |
| Cough | 7 | Prurigo | 5 |
| Cough with Dyspnœa | 3 | Herpes | 7 |
| Hoarseness | 2 | Tinea | 2 |
| Hæmoptysis | 4 | Papulous Eruptions | 12 |
| Phthisis Pulmonalis | 6 | PUERPERAL DISEASES. | |
| Dyspepsia | 7 | Mania | 2 |
| Gastrodynia | 12 | Low Puerperal Fever | 3 |
| Bilious Diarrhœa | 14 | Rhagas Papillæ | 4 |
| Colica Pictonum | 2 | Menorrhagia Lochialis | 6 |
| Hæmatemesis | 3 | INFANTILE DISEASES. | |
| Hæmorrhois | 4 | Herpes Miliaris | 7 |
| Fluor Albus | 3 | Herpes Exedens | 5 |
| Amenorrhœa | 5 | Diarrhœa | 10 |
| Menorrhagia | 6 | Aphthæ | 5 |
| Cephalalgia | 7 | | |

The excessive heat of the weather, which has for some time prevailed, has been attended, as might be expected, with a considerable aggravation of the symptoms of many diseases. Instances of Typhus have been numerous, and very generally attended attended with great determination to the head. Some patients labouring under this diſeaſe complained of confuſion of ideas, whilſt others were affected by conſiderable pain of the head; and in a few inſtances a high degree of delirium prevailed. In two puerperal caſes the patients became maniacal; one of them recovered in a few weeks; but in the other caſe the diſeaſe terminated fatally.

Cholera Morbus has been very frequent, and in ſome inſtances particularly obſtinate. The exceſſive diſcharge from the ſtomach and bowels which takes place in this diſeaſe, may at firſt view ſeem to require the uſe of powerful aſtringents to abate its violence; but the judicious practitioner is aware that conſiderable caution is neceſſary in the uſe of ſuch means. Whilſt opiates may be freely uſed to abate pain, and counteract the ſpaſmodic affections of the lower extremities, if, under the uſe of them, a SUDDEN check is given to the diſcharge from the inteſtines, it ſometimes becomes neceſſary to promote it by the uſe of ſome eccoprotic remedies. One inſtance occurred to the Reporter wherein a ſudden, though SPONTANEOUS, ceſſation of the inteſtinal diſcharge was followed by ſuch violent pain of the bowels as gave conſiderable alarm, and rendered the uſe of ſome active means neceſſary to reſtore the diſcharge, but which, as ſoon as it took place, was followed by a ceſſation of pain, and a gradual recovery to health.

